# *N*-linked glycans on the stalk of influenza virus neuraminidase promote functional tetramer formation by compensating for local hydrophobicity

**DOI:** 10.1128/jvi.00879-25

**Published:** 2025-08-18

**Authors:** Soma Saeidi, Hongquan Wan, Hyeog Kang, Jin Gao, Wells W. Wu, Tahir Malik, Robert Daniels

**Affiliations:** 1 Division of Viral Products, Center for Biologics Evaluation and Research, Food and Drug Administration 4137 , Silver Spring, Maryland, USA; 2 Facility for Biotechnology Resources, Center for Biologics Evaluation and Research, Food and Drug Administration 4137 , Silver Spring, Maryland, USA; University Medical Center Freiburg, Freiburg, Germany

**Keywords:** influenza surface antigens, glycoprotein maturation, antigen engineering, *N*-linked glycosylation, immunogenicity, recombinant vaccine antigens, surface antigen proteolysis

## Abstract

Enveloped virus surface antigens, such as influenza neuraminidase (NA), typically depend on *N-*linked glycans for assembly, trafficking in the host cell, and immune evasion. Here, we examined the function of the *N-*linked glycans on the NA stalk from H1N1 2009 pandemic (pdm09) viruses using reverse genetics coupled with a recombinant NA (rNA) analysis. Our results with the NA from A/Brisbane/02/2018 (H1N1) show that all five glycosylation sites in the stalk generally receive an *N-*linked glycan and that viral growth is largely unaffected by removing any of these sites individually. In contrast, viral growth decreased when we sequentially removed the stalk glycosylation sites, consistent with the observation that H1N1 pdm09 strains predominantly possess four or five stalk glycan sites in NA. Upon passage, the growth of the virus lacking the five stalk glycan sites significantly increased, and multiple substitutions were found that decreased the hydrophobicity near each mutated site. Reverse genetics experiments confirmed the polar stalk substitutions significantly improved viral growth and revealed an increase in the amount of NA dissociated from the virus, suggesting the stalk glycans or the hydrophobic regions protect against proteolysis. The polar substitutions also rescued the expression of a secreted recombinant NA (rNA) lacking the stalk glycan sites and significantly increased production of enzymatically active rNA that exceeded the wild-type version. These findings demonstrate that the stalk glycans promote functional NA tetramer formation by compensating for local hydrophobicity and that viral-based studies can be leveraged in the rational design of rNA antigens.

**IMPORTANCE:**

*N-*linked glycans can play critical roles in viral glycoprotein maturation and immune evasion. The influenza virus glycoprotein neuraminidase (NA) possesses multiple *N-*linked glycan sites in the enzymatic head domain and stalk region that tether it to the viral surface. This study demonstrates that the stalk *N-*linked glycans contribute to viral fitness by compensating for local hydrophobicity to enable functional NA tetramer formation. Supporting this conclusion, our results show that sequential removal of the stalk glycan sites on a NA from a recent H1N1 strain leads to decreased viral growth that rapidly recovers following passage. The increased growth coincided with stalk mutations that reduced the hydrophobicity around the stalk glycan sites. We observed similar results with a secreted recombinant NA (rNA), and the amount of functional rNA produced with the polar substitutions exceeded the wild-type rNA, illustrating how viral-based studies can assist with the rational design of rNA antigens.

## INTRODUCTION

Replication of enveloped viruses is highly dependent on the proper folding and transport of the viral glycoproteins. Influenza viruses encode for two glycoproteins, hemagglutinin (HA) and neuraminidase (NA), that perform distinct functions to facilitate viral entry and propagation (1, 2). The more abundant HA glycoprotein initiates viral entry by binding to sialic acid receptors on the cell surface (3–5). Following endocytosis, HA helps to deliver the viral genome to the host cell cytoplasm by fusing the virus and endosomal membranes (6, 7). In contrast, NA is a Ca^2+^-dependent sialidase that promotes viral movement by cleaving the local sialic acid receptors, preventing persistent HA-mediated virus binding (8–10). Properly balancing the opposing functions of these two glycoproteins is crucial for the efficient propagation of influenza viruses (11–13).

During replication, NA and HA are synthesized at the host cell endoplasmic reticulum (ER), where they mature within the ER lumen, similar to cellular glycoproteins (14–17). An integral part of the maturation process is the co- and post-translational modifications, including disulfide bond formation and *N*-linked glycan addition (18). The core *N-*linked glycans are co-translationally added to asparagine residues in the sequence Asn-X-Ser/Thr-Y, and the efficiency is influenced by the X and Y residues, with Pro at either position preventing glycosylation (19–21). Once added, the large soluble *N*-linked glycans can function to recruit lectin chaperones that assist with folding and reduce the potency of aggregation-prone regions (22–24). On viral glycoproteins, *N-*linked glycans can also contribute to immune evasion by shielding or changing epitopes recognized by antibodies in the host (25, 26). Indeed, several studies have demonstrated that *N-*linked glycan addition or removal can modulate HA and NA antigenicity in circulating influenza viruses (1, 27–29).

NA is a tetrameric, mushroom-shaped sialidase that is anchored to the viral envelope by a stalk region connected to an amphipathic *N-*terminal transmembrane (TM) domain (30–32). The sialidase activity is located in the large head domain that contains multiple *N*-linked glycan sites and intramolecular disulfide bonds (33). The stalk region also possesses several *N*-linked glycan sites as well as one or more intermolecular disulfide bonds that connect neighboring NA monomers (17, 34). Although the disulfide bonds are highly conserved, the *N*-linked glycan sites in the head and stalk regions differ by NA subtype (i.e., N1–N9) and origin of the influenza virus (35, 36).

*N-*linked glycans are added to NA during translocation into the ER lumen, where they assemble into functional tetramers by a cooperative mechanism involving the head and TM domains (10, 17, 37, 38). Previous studies using different NA subtypes have shown that the *N-*linked glycans on the head domain contribute to the folding and thermostability, which can impact NA incorporation into virions (16, 17, 35). In other work, NA tetramer formation on non-reducing SDS-PAGE (Sodium Dodecyl Sulfate-Polyacrylamide Gel Electrophoresis) gels was significantly impaired by removal of the *N-*linked glycan sites on the head, but not the stalk (17). Similarly, removal of cysteines in the NA head domain resulted in a temperature-sensitive virus, whereas removal of a conserved stalk cysteine showed minimal impact on NA activity or viral replication (34, 39). Together, these observations suggest that the co- and post-translational modifications in the short NA stalk (~50 amino acids) may contribute more to viral-specific functions (e.g., immune evasion or proteolytic susceptibility) than NA maturation.

Currently, the functions of the *N*-linked glycans on NA are not well-defined. This especially applies to those in the NA stalk, where the number of *N*-linked glycan sites increased from 4 to 5 in circulating H1N1 2009 pandemic (pdm09) viruses. Here, we systematically removed the stalk *N-*linked glycan sites in the NA from an H1N1 pdm09 virus to examine their roles. Our results show that all five glycan sites in the stalk generally receive an *N-*linked glycan and that the sequential removal of these sites results in decreased viral growth that rapidly recovers following passage. Viral genome sequencing and reverse genetics attributed the rescue in growth to polar substitutions in hydrophobic regions near the mutated stalk glycan sites, indicating the stalk *N-*linked glycans likely help to reduce the potential of deleterious interactions with these hydrophobic regions during NA assembly. In addition, the glycans or hydrophobic regions may also reduce the stalk susceptibility to proteolysis as the amount of NA dissociated from the virus increased when all the stalk glycan sites were mutated. Similar results were observed with a secreted recombinant NA (rNA), and the polar substitutions significantly increased functional rNA production compared to wild-type, illustrating how viral-based analyses can aid the design of rNA antigens.

## RESULTS

**Fig 1:**
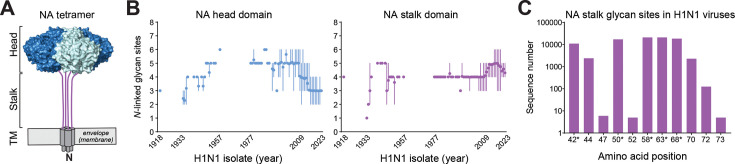
*N*-linked glycan site frequencies within the NA domains from human H1N1 viruses by year of isolation. (**A**) Diagram of an NA tetramer showing the head (residues 82–469), stalk (35–81), and transmembrane (TM) domain (residues 7–34). (**B**) Variability in the number of *N*-linked glycan sites present in the head (left panel) and stalk (right panel) domains of NA sequences from human H1N1 viruses (*n* = 21,012) by year of isolation. Data are presented as the mean (circles) with the max and min (lines) for each year. (**C**) The number of NA sequences from human H1N1 viruses (*n* = 21012) with *N*-linked glycan sites at the indicated stalk amino acid positions is displayed. Positions with fewer than five sequences were excluded. Asterisks highlight the prevalent sites in H1N1 pdm09 viruses. All analyses were performed using full-length NA sequences downloaded from the NCBI Influenza Database.

*N*-linked glycan sites on influenza NA are all located within the ectodomain that consists of the enzymatic head domain and the stalk (Fig. 1A). Analysis of the available NA sequences from human H1N1 influenza viruses revealed two temporal trends in the number of glycan sites within these two domains. The first involved a temporal increase followed by a decrease in the number of *N-*linked glycan sites in the head domain (Fig. 1B, left panel). The decrease was previously attributed to the introduction of the H1N1 pdm09 strains (35). The second trend was an increase in the number of stalk *N-*linked glycan sites from 4 to 5 that occurred between 2011 and 2012 in the H1N1 pdm09 viruses (Fig. 1B, right panel), suggesting the additional stalk glycan may have provided a selective advantage. Plotting the *N-*linked glycan sites by amino acid showed there are 8 major positions within NAs from H1N1 viruses. It also revealed that five positions (42, 50, 58, 63, and 68) predominate in the NAs from H1N1 pdm09 viruses (Fig. 1C) and that position 42 appeared around 2011, and that position 50 is no longer present in more recent strains.

**Fig 2:**
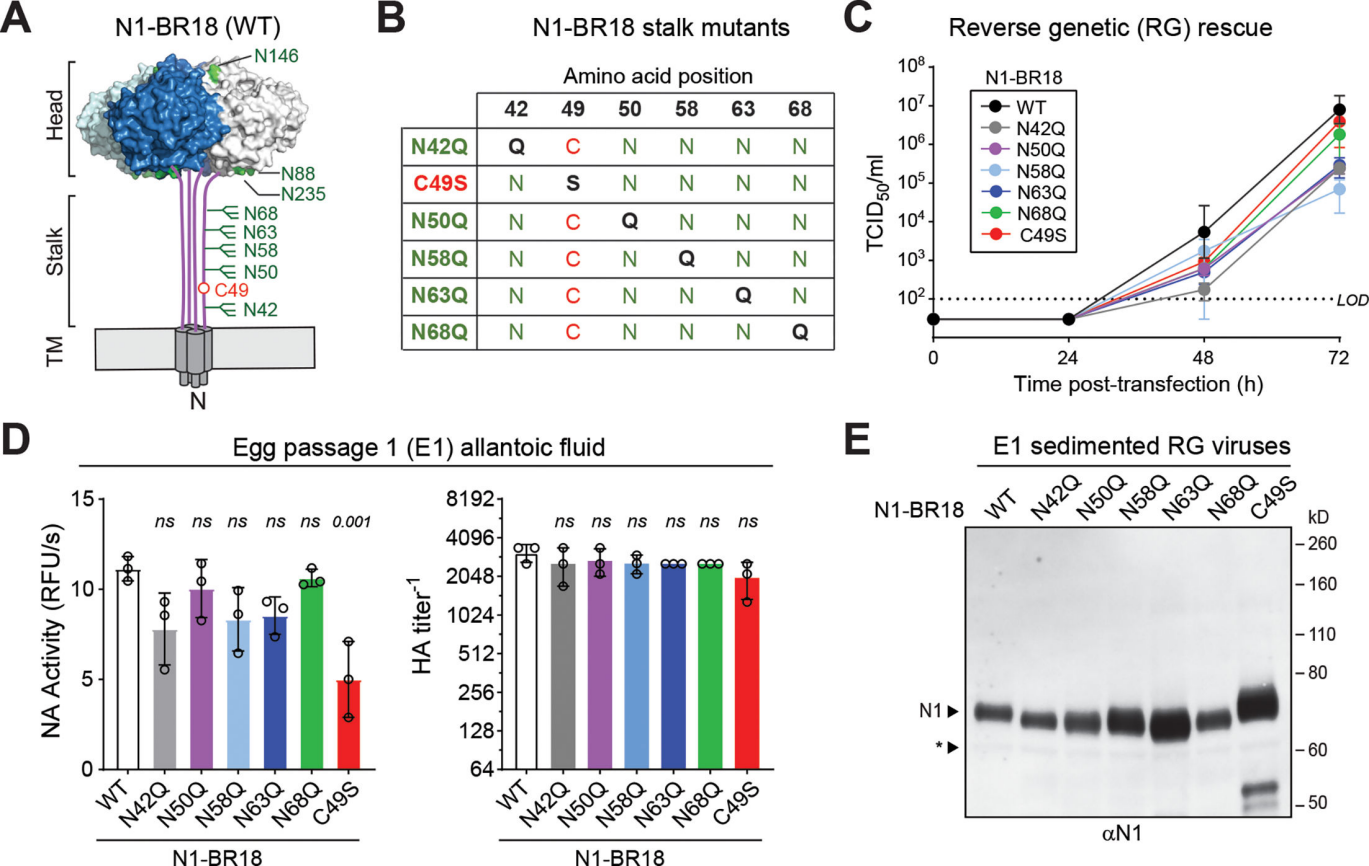
Viral growth is not impaired by removing individual *N-*linked glycan sites on the N1 stalk. (**A**) Diagram of N1-BR18 showing the location of the *N*-linked glycan site Asn residues (green) and the stalk cysteine (red). The NA head domain structure (PDB: 3NSS) was generated with Pymol. (**B**) Chart displaying the substitutions in the stalk region of the indicated N1-BR18 mutants. (**C**) Median tissue culture infectious doses (TCID_50_) of the WSN single gene reassortant viruses carrying the indicated N1-BR18 variant were measured at 24 h intervals during the reverse genetics rescue. Data are geometric means (circle) and the standard deviation (SD) from three independent experiments. The dashed line represents the limit of detection (LOD). (**D**) NA activities (left panel) and HA titers (right panel) of the rescued WSN viruses carrying the indicated N1-BR18 variants were measured after a single passage in eggs using equal allantoic fluid volumes. Each of the three independent virus rescues was passaged in a group of eggs (*n* = 3). Data are displayed as group means ± SDs. *P* values were calculated with respect to WT using a one-way ANOVA. (**E**) NA immunoblots of the indicated egg-passaged virus isolated by sedimentation and resolved by reducing SDS-PAGE. Bands corresponding to NA (N1) are indicated along with a faint background band (asterisk).

To investigate the requirement of the stalk glycans, we examined the NA (N1-BR18) from a recent H1N1 pdm09 vaccine virus (A/Brisbane/2/2018) by reverse genetics (Fig. 2A). Initially, we removed the individual stalk glycan sites in N1-BR18 by mutating the Asn (N) residues at positions 42, 50, 58, 63, and 68 to Gln (Q) residues (Fig. 2A). For comparison, we included a mutation in the stalk cysteine (C49S) that is not essential for NA function (activity) or viral growth in culture (34, 39). We then generated single-gene reassortant viruses carrying each NA with the seven backbone gene segments from the H1N1 lab strain A/WSN/33 (WSN), a neurotropic variant of the Wilson Smith strain, and measured the growth during rescue. All the individual glycan site mutants reached detectable infectious titers by 48 h post-transfection, which significantly increased by 72 h (Fig. 2C). This pattern of infectious virus production was similar to the viruses carrying N1-BR18 wild-type (WT) and C49S, suggesting removal of any individual stalk glycan site did not significantly impair the viral rescue by reverse genetics.

We then passaged each of the independently rescued viruses in groups of three eggs (e.g., three groups of three eggs per virus) and harvested the allantoic fluid 3 days later. The groups containing the stalk glycan site mutations showed variable but not significantly different NA activity levels than the WT group and all were slightly higher than the C49S mutant (Fig. 2D, left panel). Similarly, viral agglutination titers were also indiscernible from the WT group (Fig. 2D, right panel), indicating viral growth and N1-BR18 activity were not significantly affected by individually removing any of the stalk glycan sites.

Recognition of *N-*linked glycan sites can vary based on the position and sequence context (19–21). Therefore, we tested if each of the predicted stalk glycan sites on N1-BR18 receives an *N-*linked glycan by immunoblotting the passaged viruses. Compared with WT and C49S, each stalk glycan mutant displayed a subtle mobility increase consistent with the loss of an ~2.5 kDa glycan (Fig. 2E), suggesting each *N-*linked glycan site in the stalk of N1-BR18 is generally recognized.

**Fig 3:**
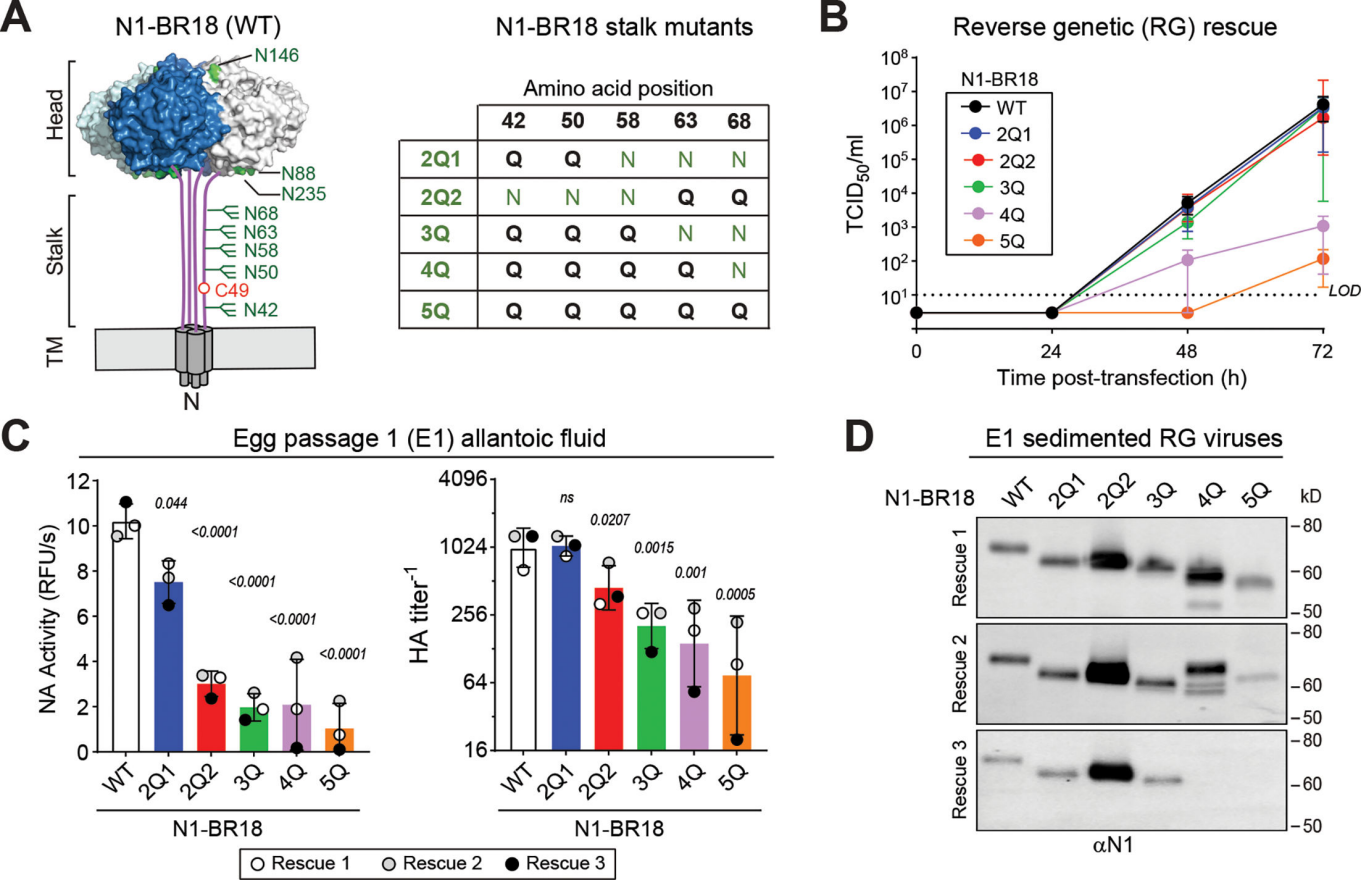
Removing multiple *N-*linked glycan sites on the N1 stalk impairs viral growth. (**A**) N1-BR18 diagram showing the *N*-linked glycan site locations (green) with a chart displaying the substitutions in the stalk glycan sites of the indicated N1-BR18 variant. (**B**) TCID_50_ of the WSN single gene reassortant viruses carrying the indicated N1-BR18 variant was measured at 24 h intervals during the reverse genetics rescue. Data are geometric means (circle) with the standard deviation (SD) from three independent experiments. The dashed line represents the LOD. (**C**) NA activities (left panel) and HA agglutination titers (right panel) of the rescued WSN viruses harboring the indicated N1-BR18 variants were measured after one passage in eggs using equal volumes. Each independent virus rescue was passaged in a group of three eggs. Data are displayed as group means ± SDs. *P* values were calculated with respect to WT using a one-way ANOVA. (**D**) NA immunoblots of the indicated viruses after a single passage in eggs. Viruses were isolated by sedimentation and resolved by reducing SDS-PAGE. Immunoblots are from the E1 passage of the three independent virus rescues.

Based on the individual stalk glycan mutant results, we created another panel of N1-BR18 mutants where either 2 (2Q1 and 2Q2), 3 (3Q), 4 (4Q), or all 5 (5Q) stalk glycan sites were removed using N to Q substitutions (Fig. 3A). During the rescue, all the WSN single-gene reassortant viruses with the different NAs generated infectious virus by 72 h post-transfection (Fig. 3B). However, viruses carrying 4 or 5 stalk glycan site mutations showed a delayed rescue, and the infectious virus amounts were substantially lower than the others. The results from the egg passage were largely similar, as both NA activity and viral agglutination titers decreased as the number of mutated NA stalk glycan sites increased (Fig. 3C). The lone exception was the virus lacking the glycan sites at positions 42 and 50 (2Q1), which produced viral agglutination titers similar to WT.

Interestingly, NA immunoblots of the passaged viruses differed between the independent experiments. In one experiment, we observed increases in N1-BR18 mobility that coincided with the number of mutated glycan sites (Fig. 3D, upper panel). In another, NA mobilities of the 4Q and 5Q mutants were slower or equivalent to the 3Q mutant (Fig. 3D, middle panel), suggesting some glycan site mutations may have reverted. In a third, the 4Q and 5Q bands were not detected (Fig. 3D, lower panel), presumably due to poor growth. Together, these results imply that viral growth is likely impaired when multiple stalk glycans on N1-BR18 are removed.

**Fig 4:**
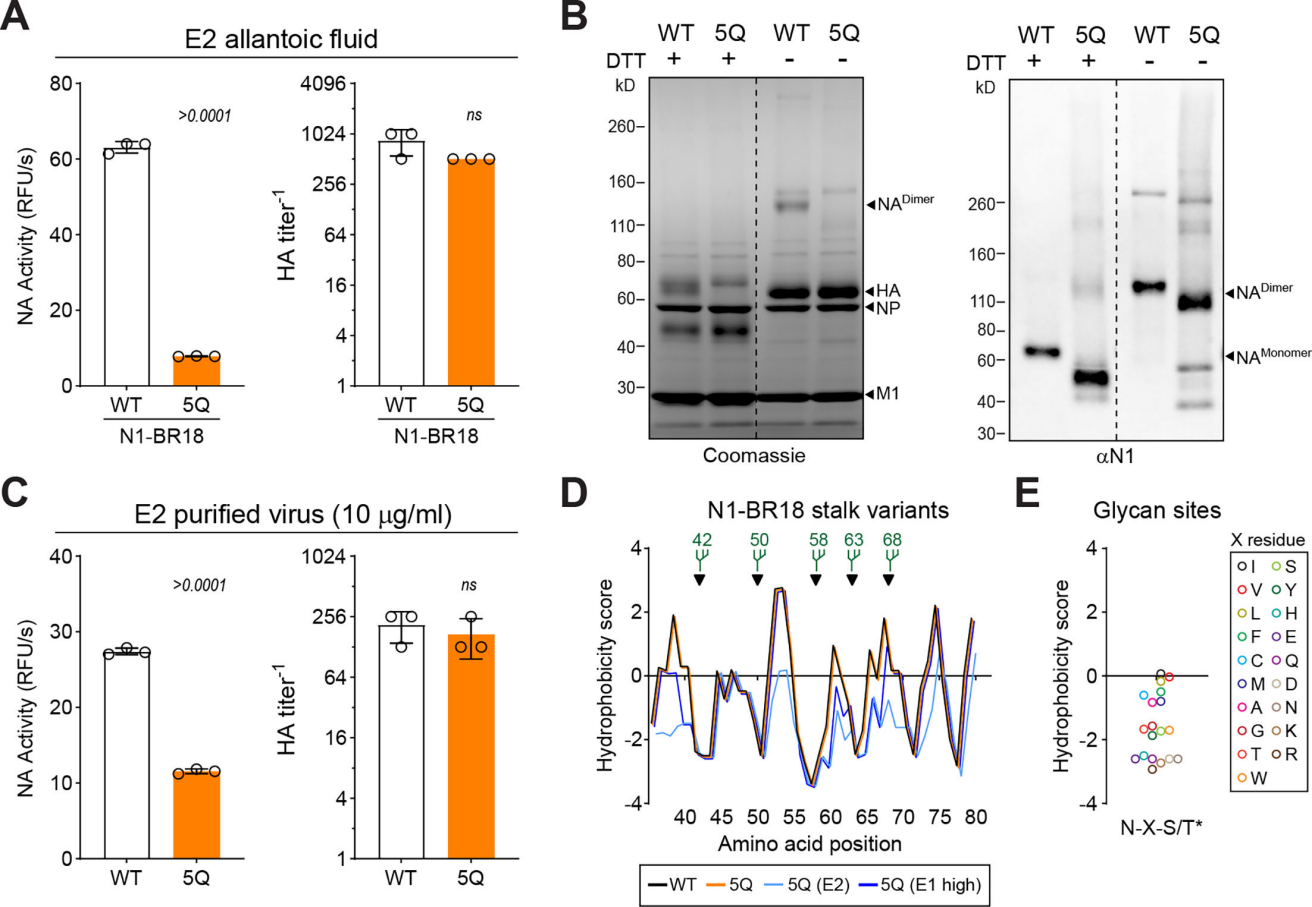
Growth of WSN carrying the N1-BR18 5Q mutant partially recovers after passaging in eggs. (**A**) NA activities (left panel) and HA agglutination titers (right panel) obtained from a second passage of the WSN viruses harboring N1-BR18 WT and 5Q in eggs. *P* values were determined by a Student *t*-test with a 95% confidence interval (CI). (**B**) Coomassie-stained SDS-PAGE gel (left panel) and an N1 immunoblot (right panel) of the WSN viruses carrying N1-BR18 WT and 5Q that were purified from the second passage in eggs. Equal amounts of total protein were resolved +/− DTT. Bands corresponding to NA, hemagglutinin (HA), nucleoprotein (NP), and matrix protein 1 (M1) are indicated. (**C**) NA activities (left panel) and HA agglutination titers (right panel) were determined using equal protein amounts (10 µg/mL) of the indicated WSN viruses purified after the second passage in eggs. *P* values are from a Student *t*-test with a 95% CI. (**D**) Kyte-Doolittle hydrophobicity profiles are shown for the stalk (residues 35–81) from N1-BR18 WT, 5Q, and the indicated 5Q variants that showed high growth after one (E1 high) or two (E2) passages in eggs. Hydrophobicity scores used a 3 amino acid sliding window, and all substitutions that comprised >5% of the viral population were included in the variant analysis (Table 1). *N*-linked glycan sites are indicated. (**E**) Kyte-Doolittle hydrophobicity scores for all potential N-X-S *N-*linked glycan sites are shown. *N-X-T values are identical with a 0.03 increase.

To gain insight into the potential function of the *N-*linked glycans on the NA stalk, we passaged a fourth independent rescue of a 5Q mutant virus that displayed low growth in E1 and the expected NA mobility shift a second time in eggs (E2). After passage, the NA activity produced by the 5Q mutant remained lower than WT, but the viral agglutination titers increased to WT levels (Fig. 4A), suggesting compensatory mutations may have been selected in the 5Q virus. We then isolated the WT and 5Q E2 viruses and resolved equal protein amounts by SDS-PAGE with the reducing reagent dithiothreitol (DTT) or without (Fig. 4B, left panel). The main difference was a single band corresponding to intermolecular disulfide bonded N1-BR18 dimers that was observed in the non-reduced sample from the WT virus but not the 5Q mutant. Immunoblot analysis showed NA was also present in the 5Q mutant virus and that it continued to display faster mobility than WT (Fig. 4B, right panel). With equal protein amounts, both viruses showed similar viral agglutination titers and the NA activity in the isolated 5Q virus remained lower than WT (Fig. 4C). We then deep sequenced the E1 and E2 passages of the 5Q virus and a fifth independent E1 passage of 5Q virus that produced unexpectedly high agglutination titers (Table 1). WT virus E1 and E2 passages were included for controls. Multiple substitutions were found in the NA of the 5Q E2 viral population and the higher growth 5Q E1 population, none of which added an *N-*linked glycan site or changed the 5Q mutations (Table 1). No mutations were found in the HA gene or either WT viral population, indicating the NA substitutions in the 5Q viruses were not related to HA changes or the use of the WSN backbone.

**TABLE 1:** NA substitutions in the indicated viral genome populations after passaging in eggs^*a*^

		Viral genome population sequenced				
		WT E1	WT E2	5Q E1 #4^*b*^	5Q E2 #4^*b*^	5Q E1 #5^*c*^
NA TM amino acid position	29	I	I	I	**T (7.3)**	I
	31	S	S	S	**P (6.5)**	S
	32	I	I	I	**T (9.9)**	I
	34	V	V	V	**A (6.8)**	V
NA Stalk amino acid position	37	S	S	S	**P (18)**	S
	38	I	I	I	**T (17)**	I
	40	I	I	I	**T (10)**	**T (40)**
	46	I	I	I	I	I
	53	V	V	V	**A (21)**	V
	54	I	I	I	**T (41)**	I
	56	Y	Y	Y	**H (65)**	**H (63)**
	61	W	W	W	**R (23)**	**R (42)**
	62	V	V	V	**A (76)**	V
	66	Y	Y	Y	**H (25)**	**H (43)**
	67	V	V	V	**A (43)**	**A (44)**
	69	I	I	I	**T (9.6)**	I
	74	F	F	F	**P (12)**	F
	79	S	S	S	**P (25)**	S
	80	V	V	V	**A (30)**	V
	83^*d*^	V	V	V	V	**A (41)**

^*a*^ All NA substitutions present in >5% of the total reads from the indicated WSN viral populations bearing N1-BR18 WT and 5Q are shown in bold. Sequencing was performed following 1 (E1) or 2 (E2) passages in eggs. The percentages of viral genomic RNA sequences encoding the substitutions in bold are displayed in parentheses.

^*b*^ Data are from E1 and E2 passages of a fourth independent 5Q rescue.

^*c*^ Data are from an E1 passage of a fifth independent 5Q rescue.

^*d*^ V83 is the second amino acid in the NA head domain.

All substitutions > 5% in the 5Q populations were in the NA stalk (residues 35–81), transmembrane domain (residues 7–34), or at a position (residue 83) next to the stalk (Table 1). Interestingly, most substitutions resulted in less hydrophobic or more polar amino acids. Based on this observation, we analyzed the hydrophobicity of the stalk region using the WT and 5Q sequences and compared those with the 5Q E2 and E1 sequences with the observed substitutions (Fig. 4D). This analysis revealed that the five glycan sites in the N1-BR18 stalk are positioned between hydrophobic peaks. It also showed that the substitutions consistently reduced the peaks around residues 40, 62, and 67, suggesting that the large hydrophilic *N-*linked glycans may help prevent deleterious interactions with these hydrophobic regions in the stalk. Supporting this hypothesis, all the recognized *N-X-S/T* glycosylation sites are hydrophilic (Fig. 4E), and the last hydrophobic peak in the stalk is followed by another conserved glycan site at position 86 in the head domain (35).

**Fig 5:**
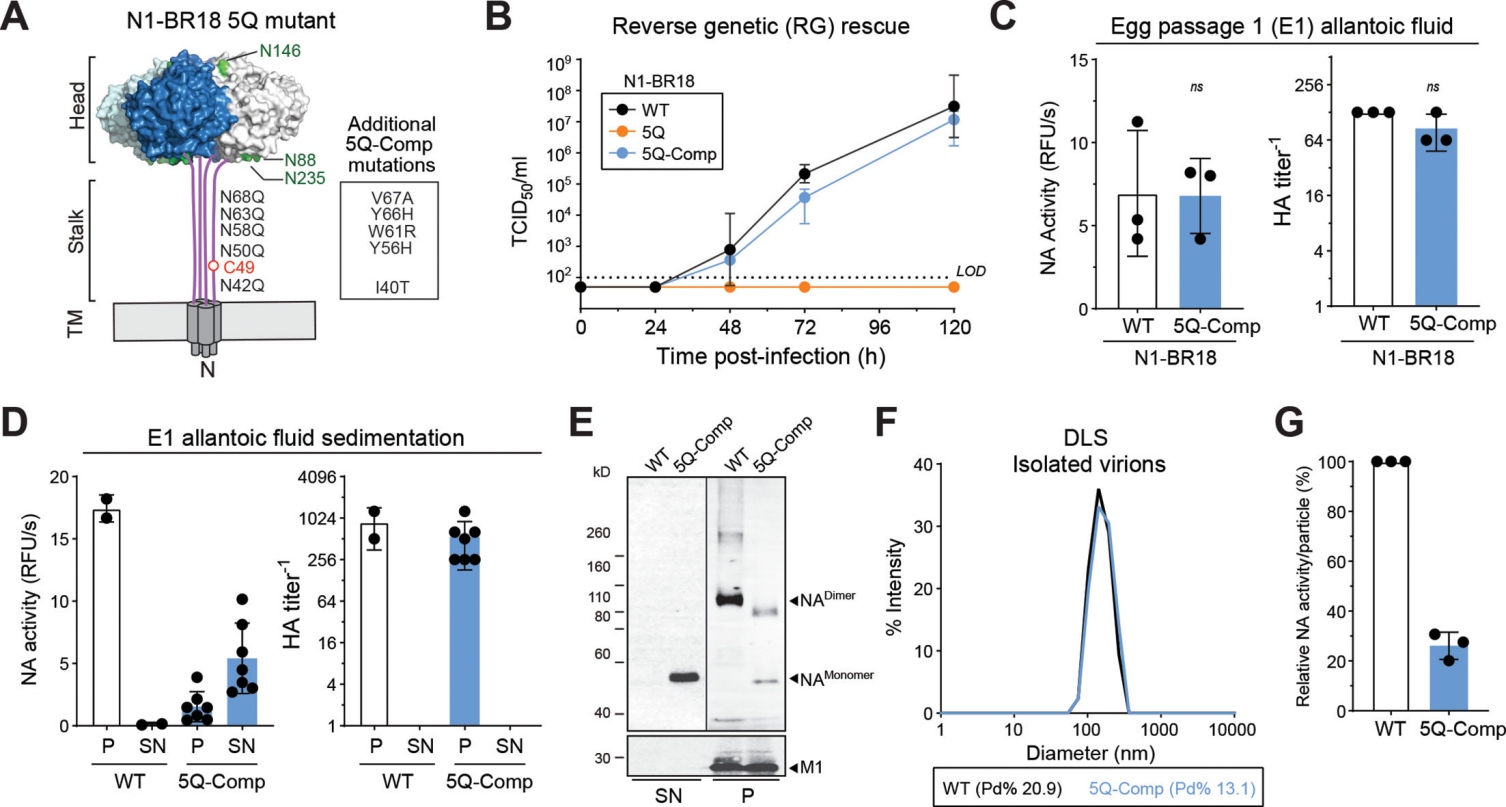
Polar stalk substitutions in N1-BR18 can compensate for *N-*linked glycan removal. (**A**) Diagram of the N1-BR18 5Q mutant showing the location of the stalk substitutions and the five additional compensatory polar substitutions that were introduced into the 5Q mutant (designated 5Q-Comp). (**B**) TCID_50_ of the WSN single gene reassortant viruses carrying the indicated N1-BR18 variant were measured at 24 h intervals during the reverse genetics rescue. Data are geometric means ± SD from three independent experiments. Dashed line represents the LOD. (**C**) NA activities (left panel) and HA agglutination titers (right panel) of the rescued WSN viruses harboring the indicated N1-BR18 variants were measured after one passage in eggs using equal volumes. Data from independent eggs are shown with the mean (bars) ± SD. (**D**) Allantoic fluid containing the indicated WSN viruses was sedimented (100,000 × *g*), and the NA activities and HA agglutination titers were measured in equal volumes of the pellet (P) and supernatant (SN). Data from independent eggs are shown with the mean (bars) ± SD. (**E**) Immunoblot of the indicated WSN viral pellet (P) and supernatant (SN) following sedimentation. Equal volumes were resolved by SDS-PAGE and probed with antisera against N1 and M1. (**F**) Diameter and polydispersity (Pd) of the indicated isolated WSN viruses were measured by dynamic light scattering. Average intensity profiles from 10 reads are displayed. (**G**) NA activities in the isolated viruses carrying N1-BR18 WT and 5Q-Comp were measured in triplicate and normalized by the number of viral particles in each sample. N1-BR18 WT activity was set to 100%.

We found five identical polar substitutions (I40T, Y56H, W61R, Y66H, and Y67A) in the stalk of the two 5Q viral populations (Table 1). Therefore, we tested if these substitutions compensated for the absence of the stalk glycans by introducing them into the 5Q sequence to create 5Q-Comp (Fig. 5A), which has the same stalk hydrophobicity profile as the 5Q (E1 high) virus (Fig. 4D). All three single-gene reassortant rescues with 5Q-Comp and WT readily produced infectious virus, whereas 5Q remained below the limit of detection (Fig. 5B), indicating the polar substitutions can compensate for the absence of the *N-*linked glycans. Supporting this conclusion, both WT and 5Q-Comp produced similar NA activity and viral agglutination titers after a single passage in eggs (Fig. 5C).

Surprisingly, a significant portion of the 5Q-Comp mutant NA activity remained in the supernatant following sedimentation, whereas the hemagglutination activity partitioned in the pellet similar to WT (Fig. 5D). To confirm these results, we immunoblotted the sedimented virus and supernatant and found that matrix protein 1 (M1) was highly enriched in the pellet of both viruses as expected (Fig. 5E). In line with the activity results, NA was observed in the supernatant of the sedimented 5Q-Comp virus, and the NA in the 5Q-Comp viral pellet showed lower intensity bands with faster mobility and some monomeric NA under non-reducing conditions. We then used dynamic and static light scattering to determine the size and concentration of the isolated virions. Both viruses showed homogeneous populations with the expected diameter (~100 nm) of an influenza virus (Fig. 5F), and we used the virus concentration to calculate that the 5Q-Comp virus possessed ~70% less NA activity than WT on average (Fig. 5G). Together, these data supported the premise that polar substitutions in the NA stalk can compensate for the absence of the *N-*linked glycans and suggest that the *N-*linked glycans or the hydrophobic regions in the stalk may reduce the susceptibility to proteolysis.

**Fig 6:**
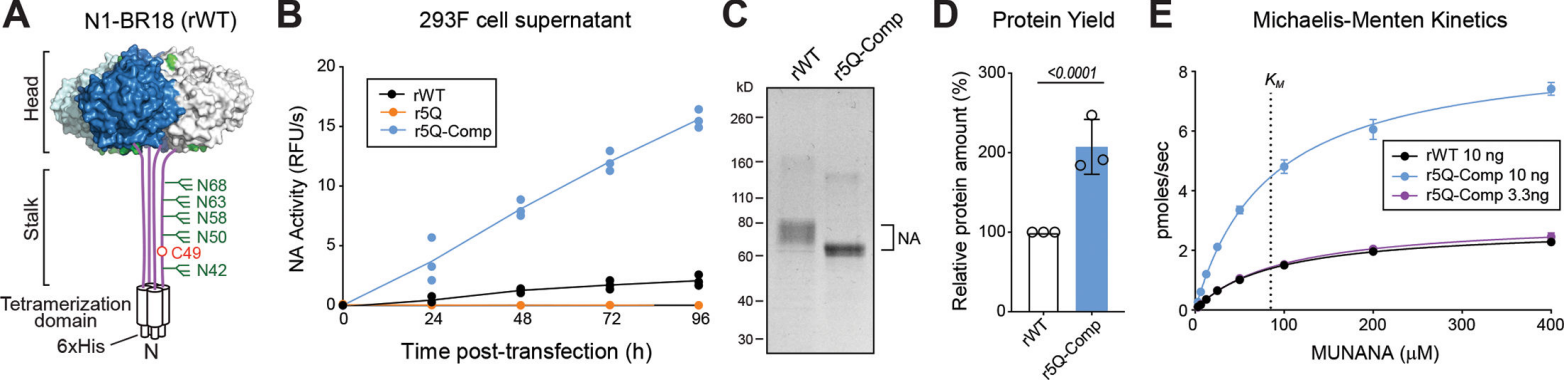
Stalk N-linked glycans and polar substitutions influence recombinant N1 production. (**A**) Diagram of the recombinant N1-BR18 wild-type (rWT) construct showing the head and stalk region connected to an *N*-terminal tetramerization domain and a His-tag for purification. Positions of the stalk *N*-linked glycan sites are in green. (**B**) NA activities were measured using equal volumes of 293F culture medium at 24 h intervals post-transfection with the indicated recombinant N1-BR18 expression plasmids. Measurements are from three independent transfections. (**C**) Coomassie-stained SDS-PAGE gel of equal amounts (2 µg) of rWT and r5Q-Comp protein isolated from the 293F culture medium at 96 h post-transfection. (**D**) Total protein yields of rWT and r5Q-Comp from three independent purifications were normalized to the rWT yield that was set to 100%. *P* value is from an unpaired Student *t*-test. (**E**) Michaelis-Menten kinetic analysis of the indicated amounts of isolated rWT and r5Q-Comp was performed in triplicate with MUNANA. Data are displayed as means ± SD. The dashed line corresponds to the calculated Michaelis Constant (*K_M_*) for the three proteins.

Finally, we reasoned that if the stalk *N-*linked glycans compensate for hydrophobic regions, the NA expressed outside a viral context would show similar results. To test this hypothesis, we compared the production of a recombinant secreted version of N1-BR18 with the wild-type stalk (rWT), the stalk glycan sites mutated (r5Q), and with the polar substitutions (r5Q-Comp) included (Fig. 6A). Initially, we monitored the expression in 293 F cells by measuring the NA activity in the culture medium at 24 h intervals. In line with the viral results, we observed increasing NA activity with rWT and r5Q-Comp but no activity was measured from r5Q (Fig. 6B). At 96 h post-transfection, we repeatedly isolated rWT and r5Q-Comp from the culture medium (Fig. 6C), but we were not able to isolate any r5Q protein, indicating removal of the stalk glycans likely resulted in misfolding, retention, and/or degradation. Interestingly, the r5Q-Comp protein yields were approximately double those for rWT (Fig. 6D), and r5Q-Comp showed a ~3-fold higher *V_max_* than rWT with the same *K_m_* (Fig. 6E), which together imply that r5Q-Comp yielded 6-fold higher functional NA. This suggests that the sialic acid binding affinity (e.g.,* K_m_*) is similar and that the higher activity (e.g.,* V_max_*) is likely due to r5Q-Comp producing a higher fraction of functional NA. Although the use of a secreted NA may have exacerbated the negative impact of the hydrophobic stalk regions, the results support the conclusion that the stalk glycans compensate for local hydrophobicity to promote functional NA tetramer formation.

## DISCUSSION

*N-*linked glycans on viral glycoproteins have been shown to perform multiple functions that range from blocking antibody epitopes to facilitating maturation and assembly within the host cell ER. Compared with the influenza HA glycoprotein, relatively few studies have examined the roles of the *N-*linked glycans on the NA glycoprotein, and these primarily focused on the sites in the enzymatic head domain that facilitate viral movement (16, 17, 27–29, 35). In this study, we used a single NA to analyze the potential functions of the multiple *N-*linked glycans in the stalk region that connects the enzymatic head domain to the viral envelope. Our results with this NA from an H1N1 pdm09 virus indicate that the stalk *N-*linked glycans promote functional NA tetramer formation by compensating for local hydrophobicity. Supporting this conclusion, removal of all the stalk *N-*linked glycan sites on NA resulted in viruses with reduced growth that accumulated polar substitutions in the stalk. Using reverse genetics, we confirmed that the polar substitutions in the stalk improved viral growth, and the resulting NA showed reduced virus association, suggesting that stalk *N-*linked glycans or the hydrophobic regions may protect against proteolysis. Similar results were observed with a secreted rNA and the polar substitutions were found to significantly increase the production of functional rNA, highlighting the potential benefits of viral-based mechanistic studies for designing rNA antigens.

Mechanistically, our data suggest that the stalk regions associate through hydrophobic domains that rely on large hydrophilic *N-*linked glycans to reduce the aggregation tendency prior to the synthesis of the neighboring NAs involved in tetramer assembly. Supporting this hypothesis, previous work has shown that secreted rNAs that contain a stalk without a stabilizing *N-*terminal tetramerization domain are enzymatically inactive and that the activity can be restored in a progressive manner by truncating the stalk (34), which effectively removes these aggregation-prone domains. In addition, NA stalks in H5N1 viruses are commonly truncated, and these truncations remove both the central hydrophobic regions and the *N-*linked glycan sites (40). Furthermore, other studies have shown that the efficiency of functional secreted rNA production can be influenced by adding stabilizing tetramerization domains with different conformations (19, 41–44) or *N-*terminal tags (45). Taken together, these findings suggest that in addition to the *N-*linked glycans, the transmembrane domain likely helps position the stalk hydrophobic regions and reduce the aggregation tendency by tethering it to the viral membrane and restricting its mobility.

The observation that viral growth defects required the removal of multiple *N*-linked glycan sites in the stalk was not unexpected for several reasons. First, NAs from H1N1 pdm09 viruses did not start encoding for the fifth glycan at position 42 until 2010 and began losing the glycan at position 50 in 2019. Second, the stalk hydrophobic regions are short and *N-*linked glycans are comparably large and flexible (46), indicating one glycan could potentially compensate for more than one region. Third, growth of the virus lacking all five stalk glycan sites improved after a single passage during one of the three reverse genetics experiments. Thus, it is possible that the viruses lacking fewer stalk glycan sites rapidly select for compensatory mutations that improve their growth characteristics.

The ability to produce functional NA without the stalk *N*-linked glycans and hydrophobic regions raises the question of why these properties have evolved. Based on the reduced viral association of the NA with the more polar stalk, we speculate that either the hydrophobic domains or the glycans may help prevent proteolysis of this likely vulnerable region. Alternatively, it could stem from an immune evasion requirement where the stalk glycans mask antigenic regions and the hydrophobic domains are required for efficient assembly of the glycosylated stalk regions. Although either function is possible, antibodies against the NA stalk have not been reported, and no structures of a complete NA antigen are available that could provide insight into the stalk assembly.

One of the more translational findings from this study was that the polar stalk substitutions substantially increased functional rNA production, likely by promoting proper tetramer formation. Recently, several studies have focused on improving influenza vaccines by using a variety of approaches to increase antibody responses against NA (43, 47–53). Many of these approaches used similar rNA antigen designs, and more recent studies have applied structure-aided design and nanoparticle coupling strategies to improve the production, immunogenicity, and stability of rNAs (44, 54, 55). Our results suggest that mechanistic studies in viruses can likely be leveraged to further optimize rNA designs, especially for regions like the stalk where no structural data exist. Although any identified mutations could change potential epitopes, increased amounts of properly folded rNA could outweigh this potential negative if the regions involved are less critical epitopes, like the stalk. Future studies are needed to determine if the stalk glycans perform similar functions in the NAs from other H1N1 viruses, as well as H3N2 and type B viruses, and serious efforts should be made to obtain structural data for entire NA antigens, as these long-ignored regions may be more informative than previously envisioned.

## MATERIALS AND METHODS

### Cells and reagents

Madin-Darby canine kidney (MDCK; CRL-2936) cells and human embryonic kidney 293T cells (HEK 293T/17; CRL-11268) were obtained from LGC Standards. Specific-Pathogen-Free (SPF) embryonated chicken eggs were purchased from Charles River Labs, and turkey red blood cells (TRBCs) from Poultry Diagnostic and Research Center (Athens, GA). Phosphate-buffered saline (PBS), pH 7.2 and pH 7.4, was obtained from KD Medical. Expi293F cells, Expi293 expression medium, Dulbecco’s modified Eagles medium (DMEM), fetal bovine serum (FBS), L-glutamine, penicillin/streptomycin (*P*/S), Novex 4-12% Tris-Glycine SDS-PAGE gels, lithium dodecyl sulfate (LDS) sample buffer, dithiothreitol (DTT), Simple Blue Stain, 6-well plates, U-bottom 96-well plates, 96-well black clear bottom plates, RNAeasy Mini Kit, and Lipofectamine 2000 transfection reagent were purchased from Thermo Fisher Scientific. Polyethylenimine (PEI) and 2’-(4-methylumbelliferyl)-α-d-*N*-acetylneuraminic acid (MUNANA) were acquired from Polysciences and Cayman Chemicals, respectively. Valproic acid was purchased from Sigma. Fluorescence Blot Blocking Buffer, 0.45 µm polyvinylidene difluoride (PVDF) membrane, and the AzureSpectra 700 goat-anti-rabbit IgG secondary were from Azure Biosystems. N1-specific rabbit MAb 11058-R001 was obtained from Sino Biological.

### NA sequence analysis

Full-length NA protein sequences from H1N1 influenza A viruses of human origin were downloaded from the Influenza Virus Resource at the National Center for Biotechnology Information (56) on January 29, 2024. Sequences were aligned and manually curated to remove mislabeled, incomplete, and truncated N1 sequences. The final data set consisted of 21,012 NA sequences from 1918 to 2023. N1 2009 pandemic-like amino acid numbering was used, and the head domain was set to begin at amino acid residue 82, and the stalk was designated as amino acid residues 35–81. Potential glycosylation sites (N-X-S/T) were identified using simple scripts and plotted by year of isolation. Stalk hydrophobicity profiles were generated using a Kyte-Doolittle analysis in CLC genomics benchtop version 10 with a window size of 3 residues to obtain higher resolution positional data.

### Virus reverse genetics, propagation, and isolation

Recombinant single-gene reassortant influenza A viruses were rescued by reverse genetics using MDCK and HEK 293T/17 cells cultured at 37°C with 5% CO_2_ and ~95% humidity in Dulbecco’s modified Eagle’s medium containing 10% fetal bovine serum and 100 U/mL of penicillin and 100 µg/mL streptomycin (57). Viruses were generated using 7 gene segments from the H1N1 strain A/WSN/1933 (WSN) and the wild-type (WT) or indicated mutant (N42Q, N50Q, N58Q, N63Q, N68Q, C49S, 2Q1, 2Q2, 3Q, 4Q, 5Q, and 5Q-Comp) of the NA gene segment from the H1N1 strain A/Brisbane/02/2018. Media was harvested at 24 h intervals for TCID_50_ measurements. Rescued viruses in cell culture medium were propagated in 10-day-old specific pathogen-free embryonated chicken eggs, and the allantoic fluid containing the viruses was clarified by centrifugation (2,000 × *g*; 5 min). For viruses isolated by sedimentation, allantoic fluid was layered on top of a sucrose cushion (25% wt/vol sucrose, PBS pH 7.2, and 1 mM CaCl_2_), sedimented (100,000 × *g*; 45 min) at 4°C, and pelleted virions were resuspended in PBS pH 7.2 containing 1 mM CaCl_2_. For purification, the resuspended virions were layered on top of a discontinuous sucrose gradient containing four 8.5 mL sucrose layers (60% wt/vol, 45% wt/vol, 30% wt/vol, and 15% wt/vol in PBS pH 7.2 and 1 mM CaCl2) and centrifuged at 100,000 × *g* for 2 h at 4°C. Fractions with a density between 30% and 50% wt/vol sucrose were pooled, mixed with 2 volumes of PBS pH 7.2 and 1 mM CaCl2, and sedimented (100,000 × *g*; 45 min). The supernatant was discarded, the sedimented virions were resuspended in 250 µL PBS pH 7.2 containing 1 mM CaCl2, and the total protein concentration was determined using a BCA (bicinchoninic acid) protein assay kit (Pierce) according to the 96-well plate protocol.

### HA assay

Viral HA titers were determined by hemagglutination assays using TRBCs. Briefly, cell culture medium from reverse genetics and allantoic fluids containing viruses were 2-fold serially diluted on 96-well U-bottom plates (Corning) using PBS pH 7.2 (50 µL/well), mixed with 50 µL of 0.5% TRBCs, and incubated at room temperature for 30 min before reading the HA titer.

### Sialidase activity assay and Michaelis-Menten kinetic analysis

NA enzymatic activity was measured by MUNANA as previously reported (52). Briefly, 25 µL of diluted viruses or rNA-containing medium was added to black 96-well clear-bottom plates and warmed to 37°C. Reactions were initiated by adding 175 µL of substrate solution (0.1 M KH_2_PO_4_ [pH 6], 1 mM CaCl_2_, and 57 µM MUNANA), heated to 37^°^C. Fluorescence (λEx: 355 nm, λEm: 450 nm) was measured by a Cytation 5 (Biotek) plate reader at 37°C for 10 min using 30 s intervals. NA activity was determined based on the slope of the early linear region in the emission versus time graph. Michaelis-Menten kinetic analysis of rNAs was performed as previously described in a 100 µL format (58). rNAs were diluted in buffer (15 mM HEPES pH 7.2, 150 mM NaCl, and 1 mM CaCl_2_), and 10 µL containing the indicated protein amounts was added to wells containing 90 µL of reaction buffer (100 mM sodium phosphate pH 6.0, 150 mM NaCl, and 1 mM CaCl_2_) with 2-fold serial dilutions of MUNANA (400 μM to 3.125 µM final concentrations in 100 µL), and the fluorescence at 37°C was measured. Slopes of the early RFU/s graphs were determined and transformed to specific activity using an umbelliferone standard curve. Once enzyme saturation was confirmed, the Michaelis-Menten kinetic analysis in GraphPad Prism 10.0 software was used to calculate the *Km* and *Vmax* for each rNA.

### Viral genome sequencing

Following the indicated passage of the rescued viruses in eggs, the viral RNA was extracted from ~500 µL of clarified allantoic fluid with the RNAeasy Mini Kit according to the manufacturer’s protocol. RNA sequencing was performed by the Facility for Biotechnology Resources (FDA). Briefly, viral genome RNA was prepared using the Illumina TruSeq stranded RNA sample prep kit without polyA enrichment, followed by sequencing using an Illumina MiSeq V3 kit according to the manufacturer’s recommendations. Sequencing data were analyzed using the FDA high-performance integrated virtual environment (HIVE) platform for amino acid substitution analysis.

### rNA expression and purification

All rNAs were secreted chimeras that contained the signal peptide from CD5 followed by a 6× His tag, the tetramerization domain from Tetrabrachion, and the entire ectodomain of N1-BR18 with the indicated mutations. Mammalian cell expression was performed by transfecting Expi293F cells cultured in Expi293 expression medium in a humidified 37°C, 8% CO_2_ incubator at 135 rpm with the indicated NA expression plasmids. Briefly, the cells were adjusted to 2 × 10^6^ cells/mL with medium; the plasmid was mixed with the medium and was added to the cell culture to a final concentration of 1 µg/mL. Cells were returned to the incubator for 15 min prior to adding PEI to a final concentration of 5 µg/mL. At ~18 h post-transfection, the culture medium was adjusted to 5 mM valproic acid, 6.5 mM sodium propionate, and 50 mM glucose. Cell culture medium was collected at 24 h intervals for NA activity measurements. rNAs were isolated from the culture medium at 72 h post-transfection by immobilized metal affinity chromatography, as previously described (45). Purified rNAs were exchanged into a pH 7 buffer (15 mM HEPES, 150 mM NaCl, and 1 mM CaCl_2_), protein concentrations were measured with a Micro BCA Protein Assay Kit (Thermo Fisher Scientific), adjusted to ~1.0 mg/mL, and aliquoted and stored at −80°C.

### SDS-PAGE and immunoblotting

Purified influenza viruses (~5 µg total viral protein for Coomassie, ~0.5 µg for immunoblots) or rNAs (~2 µg for Coomassie) were mixed with 2 × LDS sample buffer with or without 50 mM DTT, heated at ≥50^°^C for 10 min, and resolved using 4%–12% Tris-Glycine SDS-PAGE gels. Gels were either stained with simple blue or transferred to a PVDF membrane. Membranes were incubated with Fluorescence Blot Blocking Buffer, washed, probed with N1 MAb R001 (1 µg/mL overnight at 4°C prior to incubation with AzureSpectra 700 goat-anti-rabbit IgG secondary followed by washing. Coomassie-stained gels and immunoblots were imaged using an Azure 600 Imaging System. Both types of images were converted to gray scale using Adobe Photoshop 2023, and immunoblot images were subsequently inverted.

### TCID_50_ assay

Median tissue culture infectious doses (TCID_50_) per milliliter were determined using MDCK cells in 96-well plates. Initially, media containing recombinant viruses were clarified by centrifugation (2,000 × *g*; 5 min) and serially diluted 1:10 using infection medium (DMEM containing 0.1% FBS, 0.3% BSA, 1% P/S, and 1% L-gln). The media were removed from the cells; the cells were washed with PBS pH 7.4, serial viral dilutions (100 µL/well) were added to the plate and incubated for 30 min at 4°C. Viral inocula were removed, the infection medium containing 2 µg/mL TPCK trypsin was added to each well, and the plates were incubated at 37°C for 3 days. Infected wells were scored by cytopathic effects visualized under a light microscope and confirmed by an NA activity analysis. TCID_50_ values were calculated based on the method described by Reed and Muench (59).

### Dynamic light scattering (DLS) and NA activity per particle measurements

DLS measurements were performed on DynaPro NanoStar II (WYATT). Isolated virus was diluted 1:10 in buffer (25 mM MES pH 7, 150 mM NaCl, and 1 mM CaCl_2_), passed through a 0.2 µm filter, and analyzed in a 10 µL format. Data from three independent analyses were collected using 10 × 5 s intervals, and each reading set was analyzed with regularization fits in the DYNAMICS software to calculate the size distribution of the virus, polydispersity, and particle concentration (60). NA activities were measured in equal volume of the filtered virus using MUNANA, normalized by the particle concentration, and WT virus activity was set to 100%.

### Statistical analysis

Statistical analysis was performed using GraphPad Prism 10 software. All sample groups were assumed to possess Gaussian distributions and equal standard deviations. Unpaired Student *t*-tests were performed using a confidence interval (CI) of 95%. One-way ANOVAs were performed with Dunnett’s multiple comparisons test at a CI of 95%. *P* values lower than 0.05 were considered not significant, and all significant *P* values are included in the figures.

## Data Availability

Any data supporting the findings of this study are available upon request from the corresponding author.
